# Statement to an Insufficient Systematic Review on *Viscum album* L. Therapy

**DOI:** 10.1155/2020/7091039

**Published:** 2020-02-18

**Authors:** Harald Matthes, Anja Thronicke, Ralf-Dieter Hofheinz, Erik Baars, David Martin, Roman Huber, Thomas Breitkreuz, Gil Bar-Sela, Daniel Galun, Friedemann Schad

**Affiliations:** ^1^Oncological Centre, Hospital Gemeinschaftskrankenhaus Havelhöhe, Berlin, Germany; ^2^Institute of Social Medicine, Epidemiology and Health Economics and Medical Department of Gastroenterology, Infectiology and Rheumatology, Charité-Universitätsmedizin Berlin, Corporate Member of Freie Universität Berlin, Humboldt-Universität zu Berlin, Berlin Institute of Health, Berlin, Germany; ^3^Research Institute Havelhöhe, Hospital Gemeinschaftskrankenhaus Havelhöhe, Berlin, Germany; ^4^Interdisciplinary Tumour Centre Mannheim, University Medicine of Mannheim, Mannheim, Germany; ^5^Louis Bolk Institute, Bunnik, Netherlands; ^6^Gerhard Kienle Chair for Medical Theory, Integrative and Anthroposophical Medicine, Department of Human Medicine, Faculty of Health, University of Witten/Herdecke, Witten/Herdecke, Germany; ^7^Center for Complementary Medicine, Institute for Infection, Prevention and Hospital Epidemiology, University Freiburg, Freiburg, Germany; ^8^Hospital Filderklinik, Stuttgart, Germany; ^9^Hospital Paracelsus Krankenhaus, Bad Liebenzell, Germany; ^10^Emek Medical Center, Afula, Israel; ^11^Clinical Center of Serbia, Belgrade and Medical School, University of Belgrade, Belgrade, Serbia; ^12^Interdisciplinary Oncology and Palliative Care, Hospital Gemeinschaftskrankenhaus Havelhöhe, Berlin, Germany

## Abstract

**Background:**

Up to 88% of oncological patients apply complementary therapies and up to 77% apply complementary mistletoe therapy in the context of integrative oncological approaches. An evidence-based consultation of oncological health professionals regarding complementary therapies used in Germany is missing. Therefore, a new S3-Guideline for Complementary Medicine in the Treatment of Oncological Patients is under development and is anticipated to be finalized in November 2020. It will be based on evidence-based publications and systematic reviews on complementary therapies in oncology. A recently published two-part systematic review on mistletoe treatment in oncology has been reevaluated.

**Methods:**

The latest published systematic two-part review on mistletoe has been systematically proofread and checked in compliance with the Cochrane Handbook for Systematic Reviews of Intervention and the AMSTAR 2 (A MeaSurement Tool to Assess Systematic Reviews) tool.

**Results:**

The here discussed two-part review is incomplete, lacks sound accuracy including insufficient assessment of the risk of bias, and contains imprecise statements. In addition, it does not sufficiently comply with the Cochrane Handbook for Systematic Reviews of Intervention and the AMSTAR 2 tool.

**Conclusion:**

In view of the approaching release of a new guideline in the field of complementary therapies in oncology, the present statement draws attention to a lack of profound methodology of conductance of a recently released systematic review on mistletoe. In consequence, a comprehensive overview of published mistletoe studies, i.e., a meta-analysis with a sound methodology of conductance, is necessary.

## 1. Introduction

Mistletoe therapy is one of the most frequently prescribed oncological treatments in German speaking countries [1]. Up to 88% (ranging between 48.7% for colorectal and 88% for breast cancer) of oncological patients apply complementary therapies [2–6] and up to 77% (ranging between 30.6% for lung and 77.3% for breast cancer) explicitly apply complementary mistletoe therapy [7–13] in the context of integrative oncological approaches. Complementary therapies as part of integrative oncology (IO) concepts have been defined and evidence-graded in the International Society of Integrative Oncology (SIO) guideline [14] and internationally acknowledged by the American Society of Clinical Oncology in 2018 [15]. This SIO guideline considers mistletoe (grade C) for improving quality of life during and after breast cancer treatment [14]. According to the recently published updated German S3-guidelines for breast cancer, melanoma, and lung cancer, a sound professional consultation on complementary therapies which are applied in addition to standard therapies strengthen the relationship between the affected person and the physician [16–18]. However, evidence-based guidelines for physicians and health professionals on the utilization of complementary therapies in Germany are missing.

Therefore, a new German S3-Guideline for Complementary Medicine in the Treatment of Oncological Patients is under development. It will be based on evidence-based publications and systematic reviews on complementary therapies in oncology. S3 stands for the highest of three quality levels—such a guideline has undergone logic, decision, and outcome analyses as well as the assessment of the clinical relevance of scientific studies and regular reviews. The aim of the Complementary Medicine guideline is to give evidence-based and formal consented recommendations for physicians, psychologists, and other health professionals involved in oncology to ease their decision-making regarding complementary therapies. This guideline is anticipated to be finalized in November 2020.

A number of publications on mistletoe's impact on oncology have been released and two of the latest systematic reviews on mistletoe date back to 2010/2011 [19, 20]. Therefore, recently, a two-part review on mistletoe treatment in oncology was published [21, 22]. However, this two-part review suffers from important methodological flaws and has not been conducted in accordance with the Cochrane Handbook for Systematic Reviews of Intervention and the AMSTAR 2 (A MeaSurement Tool to Assess Systematic Reviews) tool [23].

In referral to the erroneous conductance of the recent systematic review, a letter to the editor was published in April 2019 criticizing numerous mistakes of this review [24]. The delineated points of criticism, however, have not adequately been answered by the review's authors in their answer in July 2019 [25]. Thus, in response to the author's answer and to the two-part review, the authors of the present statement aim at providing a thorough explanation of major points of criticism of the systematic review. It is crucial to provide the most accurate methodological reviews of data on the use of mistletoe treatment in view of the approaching complementary guideline. In the following, we will comment in an item-wise mode on unsubstantiated statements contained in the review.

## 2. Methods

### 2.1. Re-Evaluation and Analysis of the Systematic Review

The systematic review on mistletoe by Freuding et al. has been systematically re-evaluated in compliance with the Cochrane Handbook for Systematic Reviews of Intervention [26] and the AMSTAR 2 (a measurement tool to assess systematic reviews) tool [23]. To maintain objectivity, a four-person approach was independently undertaken to re-evaluate the systematic review (HM, FS, RH, and EB). Initially, all four authors independently wrote a table-based point-to-point evaluation on the systematic review which then served as the basis of the present article. In case of discrepancies of re-evaluation, they decided by consensus. The Cochrane Handbook for Systematic Reviews of Intervention has been utilized as it is the official guide for standard methods in preparing a Cochrane systematic review. We have utilized the AMSTAR 2 as a decision-making tool to evaluate the Freuding review. As healthcare evaluation is advancing, the AMSTAR 2 was developed [23] to identify the quality of systematic reviews with a view to the inclusion of real-world observational evidence and nonrandomized besides randomized controlled studies.

### 2.2. Quality Assessment and Risk of Bias Assessment

We used the Cochrane Risk of Bias tool to assess the risk of bias (high, low, or unclear) in the randomized controlled trials of the Freuding review by evaluating the risk in five domains: selection, performance, attrition, reporting, and others [27]. To maintain objectivity, a four-person approach was independently undertaken to re-evaluate the systematic review (HM, FS, RH, and EB). Initially, all four authors independently wrote a table-based point-to-point evaluation on the systematic review which then served as the basis of the present article. In case of discrepancies of re-evaluation they decided by consensus.

## 3. Results

### 3.1. Incompleteness

The review did not contain an explicit statement that the review methods were established prior to the conduct of the review and did not report on, nor, if applicable, justified any significant deviations from the protocol (see AMSTAR 2 checklist: Question 2) [23]. Furthermore, it was not stated why only randomized controlled trials (RCTs) were selected for review (see AMSTAR 2 checklist: Question 3 [23]). In fact, especially for IO with its multimodal treatment forms, various sets of evidence are reasonable. Thus, other study designs such as real-world data studies are gaining increasing importance adding value to external evidence and painting a real-world picture of the healthcare system [28]. This is explicitly mentioned in the Cochrane Handbook for Systematic Reviews of Interventions [26] and was neither used nor discussed by the authors. Including both, RCTs with their proof-of-principle concept but with inherent selection bias as well as real-world data studies, would have synergistically complemented a model of circular evidence [29]. For the selection of review questions, the authors of the review utilized the Patient, Intervention, Comparison, Outcome (PICO) model (AMSTAR 2 checklist: Question 1), which primarily focuses on therapy questions [26, 30]. They also devised inclusion and exclusion criteria according to the PICO model, however, without specifying why studies published in languages other than German or English were excluded (see AMSTAR 2 checklist: Question 4 [23]). Furthermore, the authors did not state why only reviews from 1994 onwards were included (AMSTAR 2 checklist: Question 4). Accordingly, at least two relevant randomized controlled trials (RCTs) from Salzer et al. with the endpoint “*overall survival*” which were published before 1994 were not included [31, 32]. Therefore, the review is not complete and does not justify its claim of an “*extensive overview.*” According to the AMSTAR 2 assessment tool, the quality of the review would need to be categorized as low, which means that “the review has a critical flaw and may not provide an accurate and comprehensive summary of the available studies that address the question of interest” [23].

### 3.2. Nontransparency

An important point of criticism is the nontransparency of the literature search. For example, one of the mistletoe preparations, Cefalectin®, which is described in the background section of the review, was not included as a term in the search algorithm. Secondly, the search strategy for safety concerns is missing. Therefore, the chapters “Adverse Events Regarding Mistletoe Treatment,” “Potentially Serious Adverse Events,” and “Adverse Events of Mistletoe Treatment” cannot claim comprehensiveness of safety concerns. Consequently, the selection of publications on safety may be more arbitrary than systematic and relevant publications may be missing. In addition, adverse events that were listed in electronical supplements of included publications, e.g., as in the study by Piao et al., were not included in the review. Generally, patients report adverse events (AEs) of greater than grade II, and in most cases, the physician has to actively ask the patient about the occurrence of lower-grade AEs. It is suspected that an underreporting or a nonsystematic search of AEs was performed in the review which is supported by the fact that the review does not contain information about authors of publications reporting on clinical trials who were contacted. Also, the issue “localized skin reaction” was not discussed thoroughly enough although, generally, a majority of patients experience such an AE upon subcutaneous application of mistletoe [33]. Skin reactions are indicative for immunological response and are regarded as a kind of a “desired side effect”; as such, it may have been misinterpreted in many of the reviewed studies. A discussion of this important issue as part of a risk assessment would be indispensable for a systematic review on safety aspects of mistletoe. However, this discussion was not included in the review.

In addition, the methods section lacks transparency on how the conclusion was methodologically developed concerning the results of the studies. In this respect and according to the Cochrane Handbook for Systematic Reviews of Interventions, a table of the summary of findings and a discussion including external validity (see as well our chapter “incompleteness” regarding the discussion on RCTs) are missing. Furthermore, an explorative subgroup analysis of various tumor subtypes regarding the outcome “overall survival” has not been performed. This could have been performed in conjunction with a quantitative meta-analysis which was not done by the authors (see also our criticism in the following chapter).

### 3.3. Broad Review Question vs. Narrow Review Question and Meta-Analysis

Meta-analyses augment the power of a review's statement, reduce false-negative results, and, according to the Cochrane Handbook for Systematic Reviews of Interventions, increase the “*chance of detecting a real effect as statistically significant if it exists*” [26]. It is thus unclear why the authors did not conduct a meta-analysis (the omission was only addressed as a “limitation” of the review). We had already criticized this in our letter to the editor. Yet, the author's answer that “a meta-analysis was not conducted due to the heterogeneous data from studies with mostly high risk of bias” remains unsatisfactory [25]. Firstly, the authors did not evaluate or quantify heterogeneity nor did they provide a “satisfactory explanation for, and discussion of, any heterogeneity observed in the results of the review” (AMSTAR 2 checklist, Question 14) [23]. Further, in terms of “heterogeneity,” we suggest that, if the studies included in the review had been too diverse regarding treatments with different comparators, as claimed by the authors, it is more than questionable why a systematic review with a broad review question (any mistletoe treatment in any oncological patient) was performed at all. In this case it would have been more reasonable to conduct a systematic review with a study design more restrictive as to choice of participants (e.g., certain tumor entity vs. any tumor entity), choice of intervention (i.e., mistletoe treatment alone or as an add-on treatment vs. any mistletoe treatment), and choice of the comparators (e.g., compared only to chemotherapy instead of comparison to chemotherapy, conventional therapy, no conventional treatment, or Lentinan). Otherwise, according to the Cochrane Handbook, a review with a broad study design bears the “risk of “mixing” apples and oranges (heterogeneity)” and the “interpretation may be difficult” [26]. Accordingly, it would have then been “more appropriate to prepare an Overview of Reviews,” as indicated by the Cochrane Handbook (table “advantages and disadvantages of broad versus narrow review question”) [26]. This is strongly supported by the fact that already numerous systematic reviews on mistletoe treatment exist—the authors of the systematic review mention 10 of them in the first sentence of their discussion. Among them two systematic literature reviews report a highly significant overall hazard ratio of 0.59 as to mistletoe's impact on survival with limitations of dealing with heterogeneous data driven by certain study types [34, 35]. The logical consequence of the authors' reply (see above as to heterogeneity) is that a systematic review with a broad review question would thus be obsolete.

### 3.4. Erroneous and Insufficient Assessment of Risk of Bias

According to the authors, the risks of various bias of the studies were assessed with the Cochrane Risk of Bias Tool of the Cochrane Handbook [26]. However, this assessment is to a large extent erroneously and insufficiently conducted according to the declared methods.  Table 1 illustrates the evaluation of the following errors in author's assessment.

Freuding et al. evaluated the “random sequence generation” in six studies [36–41] with a “high risk of bias.” However, the method “drawing of lots” is explicitly indicated in the Cochrane Handbook with a “low risk of bias” and needs revision by the authors of the review (see Table 1, A, Erroneous assessment of risk of bias of “Random sequence generation (RSQ)” in six studies).

In addition, blinded drawing of the lot of each patient led to their allocation to one of the treatment arms. As in these studies no randomization list existed and a single randomization was part of the enlisting process of each new patient, no risk of a prior knowledge of allocation is detectable. Therefore, according to the Cochrane Handbook, all six publications [36–41] reveal a “low risk” instead of the authors' assigned “high risk” of bias for allocation concealment [24] (see Table 1, B, Erroneous assessment of risk of bias of “Allocation concealment” in six studies).

The author attributed a “high risk” of bias for incomplete outcome data to four studies. However, in one of these four studies only 1 out of 20 patients discontinued therapy and was included in an intention-to-treat analysis which does not justify a high-risk evaluation of incomplete outcome data. Two of these four studies [42, 43] revealed only low drop-out rates (2 and 5 of a total of 220 randomized patients in the mistletoe and control group, respectively); drop outs were treated as censored cases in the fourth study, which only showed marginal effects on the total outcome as indicated by a sensitivity analysis by the same author in another publication [44]. According to the Cochrane Handbook these cases would be rated as “low risk,” not supporting the evaluation of the review's authors and thus strongly implying a need for revision (see Table 1, C, Erroneous assessment of risk of bias of “Incomplete outcome data” in five studies).

### 3.5. Nonobjective Tendency (Objective Bias)

A nonobjectivity of the review may be discussed as many of the biases were evaluated by the authors to be of “high risk” for studies with good outcome for mistletoe treatment and of low risk in studies with no positive outcome (see Table 1, D1 and D2, Inconsistent evaluation of risk of bias between studies with the same risk). For example, the risk of bias of “allocation concealment” for the Kleeberg [47] and the Piao studies [48], respectively, was inconsistently evaluated: Although in both RCTs it was not stated in detail how the randomization list was confidentially handled, the authors attribute a “low risk” for the Kleeberg study which shows no advantage of mistletoe therapy and an “unclear risk” for the Piao study with an advantage of mistletoe therapy. The same applies for another comparison between two studies regarding risk assessment of “incomplete outcome data”. The authors attribute a “low risk” to the Kleeberg study [47] which shows no impact of the mistletoe therapy compared to a “high risk” of the Tröger study which concluded an advantage of the mistletoe therapy; however, in the Kleeberg study, no estimation of an informative drop-out rate was performed (missing data in 22.2%) versus an evaluated drop-out rate (14.7%) plus sensitivity analysis on the marginal influence of the drop-out rate on the outcome results in the Tröger study. In view of these findings an objective risk assessment appears to be debatable. It is mandatory that all studies should be evaluated equally and objectively as to their risk of bias regardless of study outcome.

As to the risk of “other sources of bias,” the authors ignore the fact that essential criticism has been published [49] towards one study, the Kleeberg study [47], which they claimed to have a “low risk of bias” for “other sources of bias.” However, this existing risk of bias should have been incorporated in the study assessment and would not have justified the conclusion of a “low risk” (see Table 1, E1, Erroneous assessment of risk of bias of “Other sources of bias”).

In addition, concerning nonobjectivity of the review, the authors state in their answer to the letter to the editors (regarding the question why a meta-analysis was not performed): “To aggregate these data would insinuate a capacity of data which is not supported by the evidence” [25]. This statement suggests a rather nonobjective, biased position towards mistletoe treatment: it is the aggregation of data that serves as the basis for evidence of a systematic review and not *vice versa*. In addition, the conclusion of a systematic review can only be seen as an approximation to the evidence on the respective intervention observed. Further, if the capacity of the data of the studies were not substantial enough, any outcome of the review would consequently be without its foundation leading to a statistical dilemma.

### 3.6. Imprecise Statements

In the following, we would like to address statements made by Freuding et al. in their own review as well as statements made by them concerning potential deficits of the reviewed studies, which after thorough reading of the studies cannot be sustained. It is, therefore, anticipated that several studies have not been carefully read by the review authors.

Fourteen RCTs are reported in the review on overall survival. However, only 12 studies are listed in the review's respective table [21]. In the supplementary material regarding the outcome “overall survival,” one study serves with two results: one positive outcome of mistletoe as to nonmetastatic uterine cancer and one negative outcome of mistletoe as to the majority of included gynecological cancers [40]. Thus, as there is a discrepancy between the number of reviewed studies (*n* = 12) and the number of outcomes (*n* = 13) a misjudgment as to proportions of studies with positive as well as negative outcomes of mistletoe has to be anticipated.

**Figure 1:**
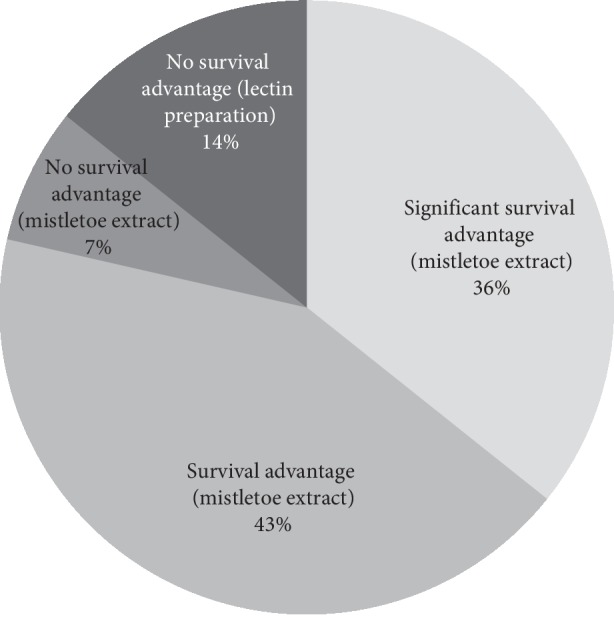
Impact of mistletoe on overall survival in oncological patients.

Based on the content of the review [21] and the inclusion of the two missed RCTs [31, 32] (see our point of criticism 1, second paragraph), we calculated that 11 of 14 studies (79%) with the outcome “overall survival” revealed a prolongation of survival and three studies (21%) did not show a prolongation of survival, of which two studies were performed with a lectin preparation and not with whole mistletoe extract preparations. In 5 out of 14 studies (36%), the survival was significant (see Figure 1).

Freuding's statement that the review does not provide any indication as to survival to prescribe mistletoe to cancer patients is not sustainable. In the conclusion, the authors of the review repeat the mistake which is explicitly mentioned in the Cochrane Handbook: “*A common mistake when there is inconclusive evidence is to confuse* ‘*no evidence of an effect' with* ‘*evidence of no effect.'. When there is inconclusive evidence, it is wrong to claim that it shows that an intervention has ‘no effect' or is ‘not different' from the control intervention. It is safer to report the data, with a confidence interval, as being compatible with either a reduction or an increase in the outcome.*” Thus, the Freuding et al. review does not only reveal a bias in its risk assessment, but also in its conclusion, both tending towards the review's negative interpretation of the effects of mistletoe.

In terms of further misleading statements, the authors state “*Further, in three studies less patients were included than was calculated in power analysis* [45, 50, 51]*. In these studies, there is a risk that no significant results were detected in spite of groups differing in reality.*” However, in two of these studies [45, 50] significant results were evaluated. Moreover, the authors state “*apart from that, in 14 studies either no power analysis was conducted or it was not reported.*” However, according to the Cochrane criteria, a study's power analysis is not a method for detecting the risk of bias but a precision criterion. We see no basis for a risk of bias due to a missing power calculation [24]. This view is supported by the Cochrane Handbook stating “*review authors should focus on the mechanisms that lead to bias rather than descriptors of studies that reflect only quality*” [26] (see Table 1, E2, Erroneous assessment of risk of bias of “Other sources of bias”).

In addition, the authors indicate that “*in at least 16 studies, there was an unclear risk of bias due to bad reporting quality in general.*” However, “bad reporting quality” is not a defined criterion for assessing the risk of bias in the Cochrane Handbook. In addition, criteria that would define a “bad reporting quality” are not provided by the authors in this review (see Table 1, E3, Erroneous assessment of risk of bias of “Other sources of bias”).

Freuding et al. attribute a multiple testing problem in one publication [50] on the impact of mistletoe on survival in pancreatic cancer [21]. Most probably, this is based on a misunderstanding of the sequential study design. A “multiple testing problem” is not attributable to this study as the only primary endpoint “overall survival” was proven in a confirmatory test (see Table 1, E4, Erroneous assessment of risk of bias of “Other sources of bias”). The consideration of multiple potential (interim) analyses was in this case covered by a group sequential study design. In addition, in their electronic supplementary material, the authors of the review state that with regard to one further study, “no control of multiple testing” was performed [52]. However, a correction for multiple testing had been performed by the authors of the respective study as explicitly stated “*For each individual analysis, the p-values of the quality-of-life scales were adjusted for multiple testing with the Bonferroni-Holm correction*” [52].

Another point of criticism of the review's authors refers to a publication [52] in which the “*palliative treatment was not described at all.*” However, the best supportive care was indeed described in this study: “*During the trial, all patients received best supportive care (BSC), which was delivered by the trial physicians. The nature of BSC was determined in the trial center; it consisted of the symptomatic treatment of pain, nausea, vomiting, and dyspepsia and was individually adapted at each of the patient's visits (in months 1, 2, 3, 6, 9, and 12)*” (see Table 1, E5, Erroneous assessment of risk of bias of “Other sources of bias”).

## 4. Discussion

The two-part review on oncological mistletoe treatment does not adhere to the criteria of a systematic review, it is incomplete, and according to the AMSTAR 2 quality assessment, it is to be categorized as of low quality because the review “*has a critical flaw and may not provide an accurate and comprehensive summary of the available studies that address the question of interest.*” Furthermore, the search strategy lacks transparency and the study validation via the Cochrane Collaboration's Tool for Assessing the Risk of Bias was applied inaccurately. Additionally, several misleading statements are found throughout the review. Moreover, certain points may raise the question, whether studies with positive outcome were rather negatively evaluated. Taking into account our points of concern regarding this review and in view of the approaching new complementary guideline, an updated comprehensive overview of published mistletoe studies, i.e., a meta-analysis with a sound methodology of conductance, has yet to be prepared. While writing this manuscript two systematic reviews with meta-analyses evaluating the association of adjuvant mistletoe with health-related quality of life [53] and with overall survival [54], respectively, are prior publishing [53] or have been published [54]. The first of these two analyses is accessible as a published pre-print version [53]. Here, 26 studies were assessed to be eligible and a significant medium-sized impact of adjuvant mistletoe extracts on the quality of life (*d* = 0.61; 95% CI: 0.41–0.81; *p* < 0.0001) was observed [53]. The results of the latter analysis (*n* = 32 studies) indicate adjuvant mistletoe Iscador treatment being associated with a better survival (HR = 0.59; CI: 0.53 to 0.65, *p* < 0.0001) in oncological patients [54]. In the Loef and Walach analysis [53], the moderators of heterogeneity could not finally be clarified due to assumedly multiple interactions between different moderators which, according to the authors, would not be detectable with a maximum set of 30 studies [53]. Therefore, by adding further upcoming mistletoe studies in future meta-analyses will help to shed light on interplaying heterogeneity moderators and on mistletoe's impact. The results of a prospective randomized placebo-controlled phase III study on mistletoe therapy in primary and recurrent inoperable pancreatic cancer (MISTRAL, EudraCT Number 2014-004552-64; to be completed by June 2021; primary outcome survival) of a randomized open-label, active-controlled, prospective, multinational phase III study on the intravesical mistletoe extract in superficial bladder cancer with tumor recurrence (EudraCT Number 2013-003446-16; to be completed by June 2021; primary outcome tumor recurrence) and of a prospective randomized multinational safety and efficacy study on subcutaneous mistletoe in the palliative therapy of pancreatic cancer patients (PALM-Pan, EudraCT number 2014-002386-30, primary outcomes overall survival and fatigue) are to be awaited.

Even though the present statement is a necessary complement to the recently published evaluation on mistletoe's impact, it cannot replace the comprehensiveness of a systematic review and this can be seen as a limitation. However, further updated and high-quality systematic reviews and meta-analyses including the works of Ostermann et al. and Loef and Walach are on the way joining the queue of growing mistletoe's clinical body of evidence.

## 5. Conclusions

The here-discussed systematic review does not allow to draw relevant conclusions to the impact of mistletoe treatment as they are not sufficiently substantiated and, therefore, lack justification. Thus, the discussed review would either need to be thoroughly revised or considered to be withdrawn from the journal in which it was published. In consequence, a comprehensive updated overview of published mistletoe studies, i.e., a meta-analysis with a sound methodology of conductance, is necessary.

## Figures and Tables

**Table 1:** Corrections as to risk of bias assessment.

	Issue	Relevant studies	Risk	Review's assessment [21]	Corrected evaluation according to Cochrane Handbook^*∗*^
A	Erroneous assessment of risk of bias of “random sequence generation (RSQ)	[36–41]	Random sequence generation	High risk	Low risk

B	Erroneous assessment of risk of bias of “allocation concealment” in six studies	[36–41]	Premature knowledge of allocation	High risk	Low risk

C	Erroneous assessment of risk of bias of “incomplete outcome data”	[42–46]	Incomplete outcome data	High risk	High risk of bias is not justified

D1	Inconsistent evaluation of risk of bias between studies with the same risk	[47] vs. [48]	Allocation concealment	Low risk, for a study with no advantage of mistletoe [47] vs. high risk, for a study with advantage of mistletoe [48]	Equal assessment of risks

D2	Inconsistent evaluation of risk of bias between studies with the same risk	[47] vs. [44]	Incomplete outcome data	Low risk, for a study with no advantage of mistletoe [47] vs. high risk, for a study with advantage of mistletoe [44]	Equal assessment of risks

E1	Erroneous assessment of risk of bias of “other sources of bias”	[47]	Other source of bias	Low risk, for a study with no advantage of mistletoe	High risk, as essential criticism has been published [49] towards the Kleeberg publication

E2	Erroneous assessment of risk of bias of “other sources of bias”	[45, 50, 51]	Other source of bias	High risk	Low risk, a in two of the studies [45, 50] significant results were evaluated

E3	Erroneous assessment of risk of bias of “other sources of bias”	“*at least 16 studies*”	Other source of bias	Unclear risk	Low risk, as “bad reporting quality” is not a defined criterion for assessing the risk of bias according to the Cochrane Handbook^*∗*^

E4	Erroneous assessment of risk of bias of “other sources of bias”		Other source of bias	High risk	Low risk, as a “multiple testing problem” is not attributable to this study as the only primary endpoint “overall survival” has been proven in a confirmatory test

E5	Erroneous assessment of risk of bias of “other sources of bias”	[52]	Other source of bias	High risk	Low risk, as the individual best supportive care was described in this study

^*∗*^Cochrane Handbook for Systematic Review of Interventions [26].

## Data Availability

The data used to support the findings of this study are available from the corresponding author upon request.
